# Inclusive engagement for equitable resilience: community case study insights

**DOI:** 10.1088/2515-7620/ad9242

**Published:** 2024-12-13

**Authors:** Emily Eisenhauer, Keely Maxwell, Brittany Kiessling, Siena Henson, Marissa Matsler, Raven Nee, Maureen Shacklette, Meridith Fry, Susan Julius

**Affiliations:** 1 U.S. Environmental Protection Agency Office of Research and Development, Washington, DC, United States of America; 2 U.S. Environmental Protection Agency Office of Research and Development, Research Triangle Park, NC, United States of America; 3 Oak Ridge Institute for Science and Education Research Participant at U.S. Environmental Protection Agency Office of Research and Development, Washington, DC, United States of America; 4 U.S. Environmental Protection Agency Office of Research and Development, Corvallis, OR, United States of America; 5 Oak Ridge Associated Universities Participant at U.S. Environmental Protection Agency Office of Research and Development, Washington, DC, United States of America

**Keywords:** resilience, equity, climate change, community engagement, social science, vulnerability

## Abstract

Incorporating equity into climate resilience planning, especially through participatory processes, is important to adequately address social vulnerability and avoid reproducing inequities. Recent analyses of resilience and adaptation plans in the United States suggest that there is increasing attention on equity and justice, but a wide variation in how it is being incorporated and implemented. Available studies of resilience planning are limited by their focus on larger urban areas and on plan contents. This research contributes a qualitative analysis of participatory engagement for resilience planning in smaller cities and rural areas. It presents findings from community case studies used as part of human-centered design research to develop an equitable resilience planning tool. Materials from the tool were used to conduct participatory engagement activities including storytelling, mapping, and brainstorming actions that elicited community members’ experiences with hazards and disasters and ideas for equitable resilience actions. Themes that emerged from the qualitative analysis of the workshop discussions were: community members’ include both environmental and social concerns in addressing resilience, challenges associated with social vulnerability framing, the underlying social systems that perpetuate inequities, recognizing different types of trauma, the power of convening, and challenges with sustaining engagement without dedicated resources. This article provides insights that inform efforts to better incorporate equity into resilience planning and advance the study of equitable resilience.

With the intensification of climate change impacts and related disasters, an increasing number of US cities are undertaking resilience planning. Equity is an integral part of resilience, as communities with higher levels of vulnerability tend to suffer greater damage in disasters and take longer to recover (Dhakal and Zhang 2023). However, climate resilience, adaptation, and mitigation plans have been critiqued for their limited inclusion of equity, inclusion, climate justice, environmental justice, and social vulnerability (Shi *et al* 2016, Meerow and Newell 2019). In the United States, city and county-level plans are beginning to incorporate concepts of equity and justice (Schrock *et al* 2015, Chu and Cannon 2021, Fiack *et al* 2021, Diezmartínez and Short Gianotti 2022). However, the degree to which they are addressed differs. One examination of climate action plans in large U.S. cities found that although social equity was mentioned in nearly 90 percent of plans, it received significant attention in only a few (Schrock *et al* 2015). Further examination is needed into how best to incorporate equity and justice into planning for disasters and climate change (VanBuskirk and Mullenbach 2024). In this paper we share observations from three case studies using the Equitable Resilience Builder, an EPA resource for equitable resilience planning, and offer insights into aspects of community engagement that support increasing equity in resilience planning.

Resilience is traditionally thought of as being able to withstand and recover rapidly from disruptions, and now is increasingly incorporating adaptation to changing conditions as well (USGCRP 2023). Equitable resilience is ‘a form of human-environmental resilience which takes into account issues of social vulnerability and differentiated access to power, knowledge, and resources,’ taking seriously ‘people’s own perception of their position within their human environmental system’ and the need for changes to social systems so that inequities are not perpetuated (Matin *et al* 2018, p.198). Currently available resilience plans, which include those specifically named as such and others that address climate impacts and disasters such as climate adaptation plans and hazard mitigation plans, vary widely in how and whether they address justice and equity, social vulnerability, and social systems. The tripartite approach of distributional (i.e., how the benefits and burdens of a changing climate vary across local populations or geographic areas), procedural (i.e. who was included in creating the plan) and recognitional (i.e. who are the affected people) justice stemming from the environmental justice literature (Bullard 1994, Marino *et al* 2023) is useful for analyzing these differences. Meta-analyses of resilience plans reveal that distributional justice is most common in plan content, with attention to the distribution of resources, amenities, services, and climate impacts. While many plans also specify public consultation steps taken as reflective of procedural justice to a greater or lesser extent, substantial consideration of recognitional justice, or the social identities, histories, values, and aspirations of those especially affected by climate change, is least common (Meerow *et al* 2019, Chu and Cannon 2021, Fiack *et al* 2021, Raub *et al* 2023).

It is well established that how and why people experience disasters and a changing climate as they do is cleaved along the lines of race, ethnicity, gender, sexuality, occupation, citizenship, income, and other social identities and geographies, and is reflective of historic and contemporary patterns of inequality and systems of oppression (Jacobs 2019, Thomas *et al* 2019, Hayden *et al* 2023, Marino *et al* 2023). Most plans that discuss such differential vulnerability rely on social vulnerability analysis, typically a quantitative analysis using available population and location data such as the U.S. Census (Maxwell *et al* in review). This analysis mostly lacks consideration of historical or societal factors such as systemic racism (VanBuskirk and Mullenbach 2024), treating vulnerability as a static condition inherent to population groups rather than as a condition produced by societal factors (Simon and Dooling 2013). When plans do acknowledge that societal forces contribute to differential hazard vulnerability, it is primarily at the surface level of the distribution of climate impacts in relation to race and poverty. Most do not have a deeper reckoning with root causes of social vulnerability, intersectional identities, and what it truly would mean to achieve equitable and just climate solutions (Meerow *et al* 2019, Chu and Cannon 2021, Diezmartínez and Short Gianotti 2022, Raub *et al* 2023). As Raub *et al* (2023) state, ‘resilience planning ignores the social structures and institutional processes that perpetuate systemic inequities’ (p. 47). This failure runs the risk that resultant interventions actually worsen social inequalities as related to the distribution of climate impacts, and the negative consequences of adaptation, resilience, and mitigation projects themselves (Zoll *et al* 2023, Marino *et al* 2023, USGCRP 2023). Recent studies show demonstrable inequalities in the benefits and burdens of green infrastructure projects, for example (Hoover *et al* 2021, Zoll 2021, Zuniga-Teran *et al* 2021, Hoover *et al* 2023).

Such knowledge and approaches from the social sciences on vulnerability, resilience and equity are necessary yet underutilized in the design of climate solutions (Devine-Wright *et al* 2022, Hackmann *et al* 2014, Schipper *et al* 2021). The social sciences provide contextual understandings of how social and environmental systems interact in specific geographies, cultures, and populations, to shape climate drivers, hazards, vulnerabilities, and impacts. For local climate adaptation and resilience planning, it means incorporating evidence and data from psychology, anthropology, sociology, and other disciplines when developing plans. It also means using alternative frameworks stemming from the social sciences, particularly from historically underrepresented voices within the scientific academy, for defining the problem space and the solution space, such as intersectionality (Crenshaw 1989, Amorim-Maia *et al* 2022), Black feminist approaches (Jacobs 2019), Queer studies (Goldsmith *et al* 2022), Indigenous studies (Whyte 2017), and environmental justice (Bullard 1994, Lee 2021).

Achieving climate justice entails transformative approaches that reckon with the power structures, epistemologies, historical marginalization and dispossession, resource flows, and environmental and health burdens that underlie distributional, procedural, and recognitional justice (Jacobs 2019, Meerow *et al* 2019, Shi and Moser 2021, Amorim-Maia *et al* 2022, Marino *et al* 2023). Identifying potential avenues for such approaches in communities requires planning processes and activities that incorporate all three dimensions of environmental justice, centering on the inclusion of community members most likely to be directly impacted and suffer the most harm from climate impacts and other hazards and disasters. Few studies of resilience planning focus on examining the procedural or recognitional aspects of planning processes. One such review of participation in coastal adaptation and hazard mitigation planning in the U.S. (Mezei *et al* 2023) identified challenges to participation including: local governance capacity, funding mechanisms, and lack of bilateral knowledge transfer with communities. Urban planning scholars have been developing and studying neighborhood- and municipal-scale methods that increase inclusive participation in planning activities for the past 30 years (Healey 1992, Innes and Booher 2010). Increasingly these methods include principles of justice, diversity and participation in order to improve quality of life for all (Fainstein 2010). For example, equity planning centers the needs of the vulnerable by asking every urban policy to first ‘increase the choices for those who have the fewest.’ Equity planning also recognizes the power inherent in the role of the planner; while planners are primarily implementers, they often have power over the process of deciding who is at the decision-making table in the first place (Zapata and Bates 2015). Therefore, there is an opportunity to empower planners to set a table that is more representative and inclusive of local communities.

Further advancement in participatory processes have focused on moving beyond simple community involvement to actual integration of community knowledge and perspectives in decision-making. This requires more than just giving community members a seat at the table; it involves active listening and *centering* community knowledge in planning decisions (Jacobs 2019). EPA designed the Equitable Resilience Builder (ERB) as a support tool for resilience practitioners and others who want to incorporate equity more centrally in their planning (Fry *et al* 2023). The tool utilizes participatory planning principles and techniques such as participatory mapping, storytelling, and action brainstorming to help communities identify barriers to resilience and equity and actions to address them. This is accomplished by intentionally creating space for discussion of the underlying social systems that produce vulnerability and ways that they can be transformed to meaningful and equitably increase resilience.

Below we share observations from case studies where we piloted this approach in several communities, using materials from the ERB to convene workshops together with community partners for the purpose of identifying key resilience and equity issues and actions to address them. Namely, we offer insights into the ways that participatory activities such as storytelling and facilitated discussions about equity can go beyond traditional hazards and vulnerability data analysis to generate discussions about underlying systemic issues and ideas for actions that center equity in resilience. We also highlight lessons learned about limitations and additional factors needed for the success of resilience planning efforts such as understanding community trauma and capacity for sustained engagement.

## Methods

The ERB was developed using human-centered design (HCD), a systematic process for gathering data about user needs, generating empathy for prospective audiences, identifying problems, and designing solutions (Gunn *et al* 2013, Baxter *et al* 2015, Lab at OPM 2018a, 2018b). The process is inherently collaborative and iterative, moving through phases of discovery, synthesis, ideation, prototyping, and testing. At each step we engaged with local government planners and officials, resilience and equity practitioners, and non-profit community organizations (for more about the process, see Fry *et al* 2023). As part of the prototyping and testing phases, the research team conducted three case studies to test and refine ERB materials, concepts, and approaches with communities.

The case studies took place between the summer of 2022 and spring 2023. Locations were selected in collaboration with EPA programs and regional offices that saw a need and an opportunity for assisting communities with resilience planning. The three case studies involved the following communities, (1) the Lower Grand River Organization of Watersheds, in Grand Rapids, Michigan, (2) Barnwell, South Carolina and Waynesboro, Georgia, and (3) Buffalo, New York.

Each community collaborated with the ERB team to customize and apply ERB materials for their specific priorities:

- The Lower Grand River Organization of Watersheds (LGROW) has been an Urban Waters Federal Partnership (UWFP) location since 2011. LGROW works in Grand Rapids, Michigan, to understand, protect, and improve the natural resources of the Lower Grand River watershed for all to enjoy. In response to increased climate change impacts, LGROW began to create a Watershed Resilience Action Plan that would provide recommendations to local governments and communities on actions to enhance resilience. LGROW and partners saw an opportunity to use ERB to increase engagement with historically underserved communities in the watershed and develop recommendations for the Watershed Resilience Action Plan that included equity considerations.
- Barnwell and Waynesboro are two communities located in the Central Savannah River Area. This region is exposed to a Superfund site with high concentrations of chemical and radiological pollution. EPA Region 4, in collaboration with faith-based community partner the Imani Group, identified the need to develop emergency preparedness and climate resilience strategies that would address disparities in aid and available resources.
- For the community in Buffalo, NY, EPA Region 2 and partners, including New York State Department of State (NY DOS), and FEMA Region 2, sought to apply ERB to develop an understanding of the intersectional impacts of COVID-19 on underserved communities and ways to support community recovery. This approach was particularly important to the partner group because Buffalo, NY has a legacy of industrial contamination and post-industrial disinvestment. These factors have negatively impacted lower-income residents in the East Side disproportionately compared to wealthier neighborhoods in the city.

Each case study was led by a ‘core team’ of EPA staff, local organizations, and experts in climate adaptation planning contracted by EPA^6^ to guide and facilitate the process. The core teams selected the ERB activities that would help the local organizations achieve their goals and customized the ERB materials for the particular objectives and timing of each workshop. The teams held regular meetings for three to four months to plan the workshop logistics and agendas, recruit participants, and customize materials. Each case study culminated in workshops with community residents, community-based organizations, faith communities, and local officials. In Grand Rapids, three day-long workshops were held in three different sub-watersheds with a total of 89 participants. In Barnwell and Waynesboro, two half-day workshops were held two weeks apart with a total of 54 participants. In the case of Buffalo, NY, the initial workshop plan was set aside due to a series of traumatic events, including the racially motivated mass shooting in East Buffalo. In an effort to focus on community recovery and healing, the EPA and partner team first offered a training in trauma-informed engagement to 11 local organizations (state and local governments, NGOs, and local universities). The training, and further discussions, led to a day-long resource mapping workshop with 24 participants which focused on identifying existing resources and resource gaps while brainstorming how to better connect technical and funding sources with community needs.

Although each of the case studies had unique objectives, the workshops all shared the intention of bringing together community members to share experiences and concerns and propose ideas for increasing resilience and equity in the community. The workshop activities were designed to provide a forum for discussion and documenting of critical issues that community members felt were barriers to equitable resilience, as well as building connections and relationships among workshop participants. This qualitative, local knowledge fills important gaps in the data and information available to planners and practitioners about resilience challenges and inequities.

**Figure 1.**
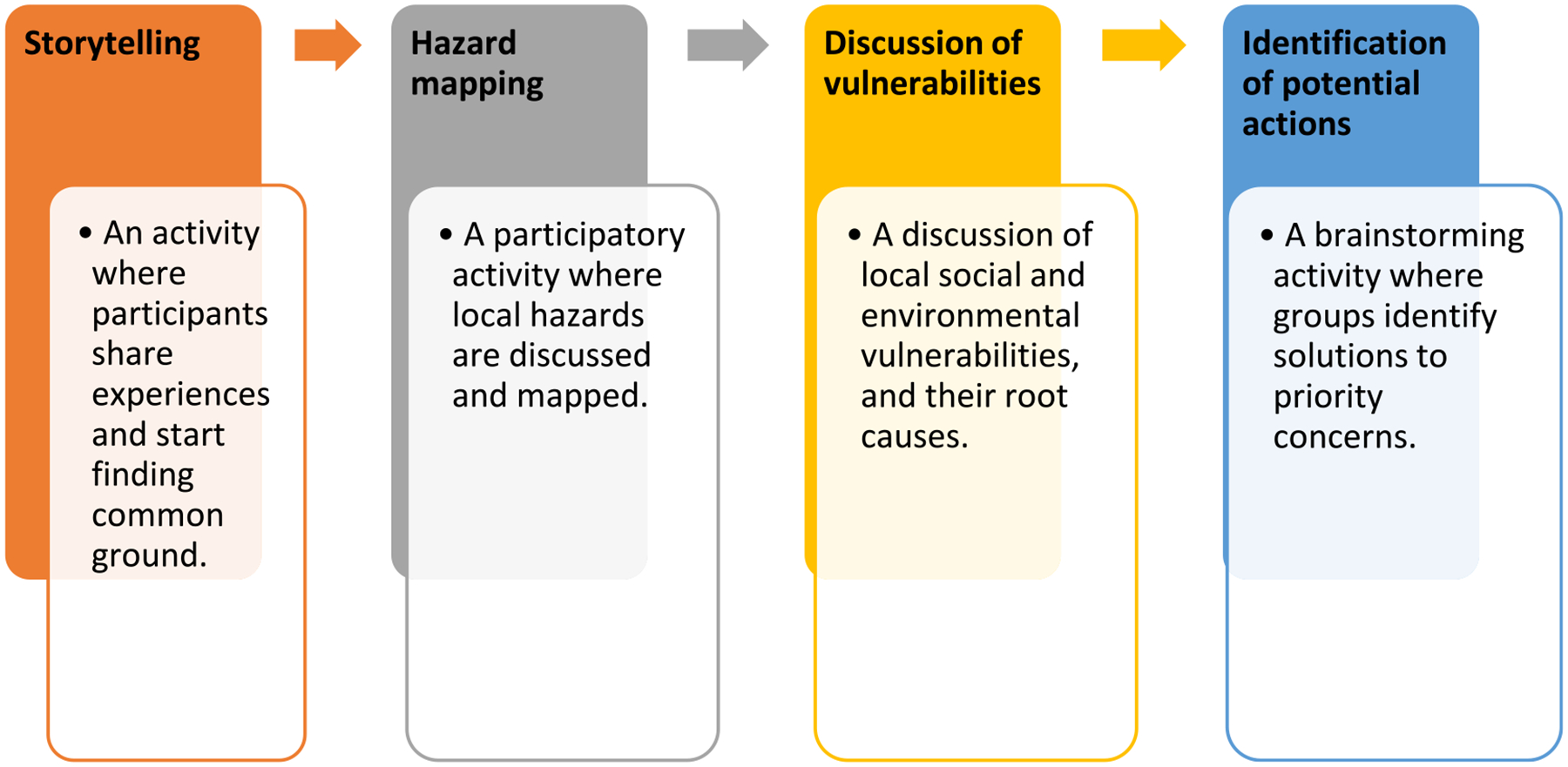
The general flow of workshop activities.

**Figure 2.**
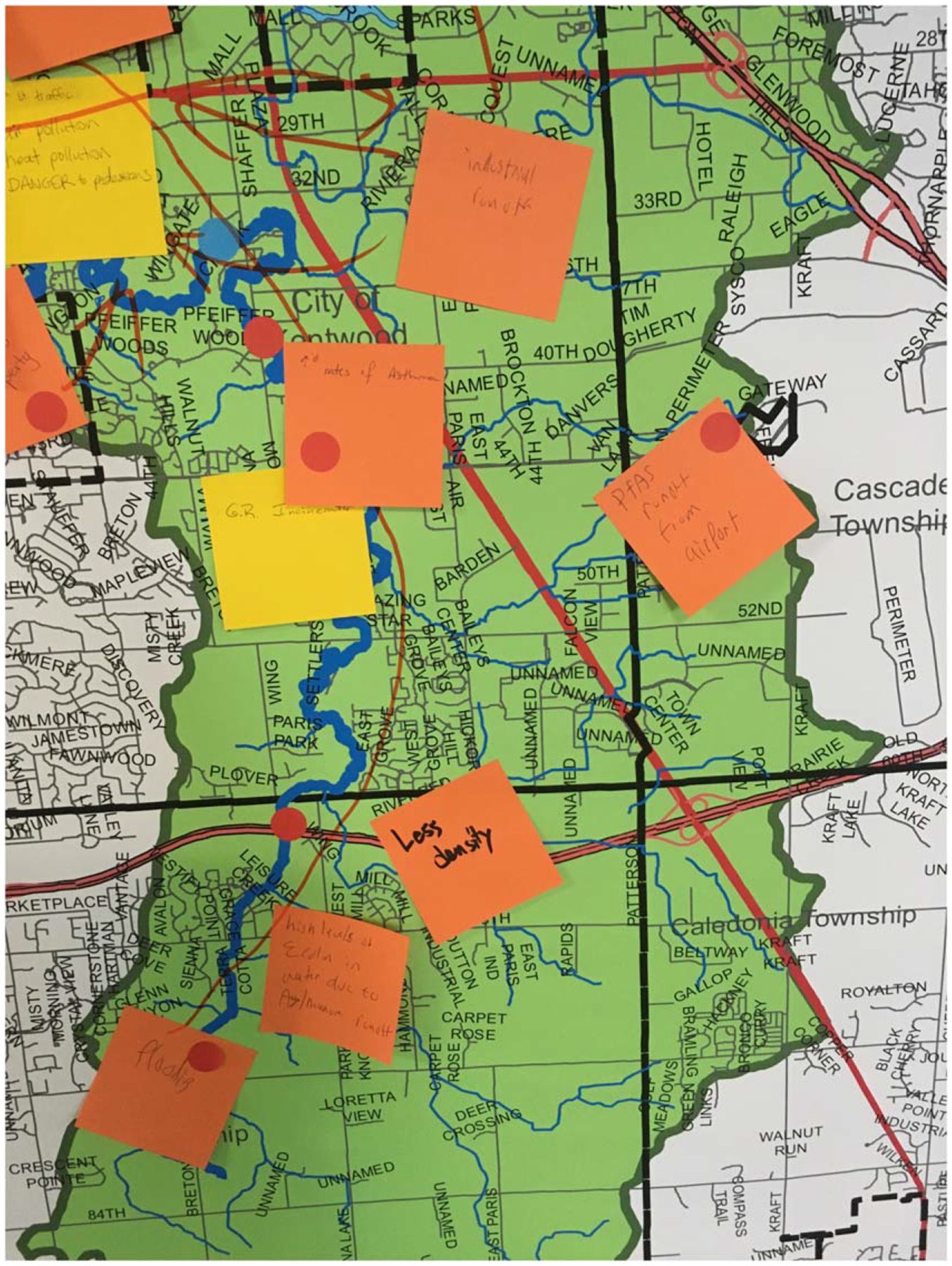
A hazard map created during a Grand Rapids, Michigan case study workshop.

During the workshops, facilitators (which included EPA staff, contractors, and community partners) guided participants through the customized ERB activities, and the workshops generally followed a similar structure (see Figure 1) that included storytelling, participatory mapping, discussions of vulnerabilities, and identification of potential actions. The storytelling activity prompted participants, in small groups of three, to share an experience with a hazard, disaster, or threat by describing what happened, how they were impacted, what happened afterward, and what helped them during the experience. The small group size allowed everyone the opportunity to share a story without having to share with the entire group and allowed participants to have more personal interaction with one each another. After the small group sharing all the participants got back together and discussed the types of hazards and impacts the community had experienced. Next, the participatory mapping activity prompted participants to identify current hazards or locations particularly vulnerable to climate impacts by placing sticky notes on large maps hung on the walls (see Figure 2). In some cases, potential future hazards were also discussed, and community assets were identified. Finally, voting with a show of hands was used to select four to five issues that participants wanted to discuss in breakout groups for brainstorming actions.

The research team documented the case studies by taking detailed notes during and immediately following the workshop proceedings. Participants were informed that notes would be taken during the workshops for research purposes, but no individually identifiable data would be collected, and given the chance to decline participation. The notes followed a standardized notetaking template to ensure notes were systematically recorded. Other materials were also generated during the workshops, including flip charts and worksheets. These additional materials contained insights collected from each of the workshop participants and were used to develop the key takeaways for each case study.

Following the workshops, the core teams met to reflect on the process and synthesize outcomes. Reflection discussions involved topics such as what actions took place since the workshops, feedback that was received from participants, how the process was helpful for meeting community partner goals, future needs, and how EPA can continue to support this work. These reflection discussions were important for correcting biases from the research team such as pre-conceived notions about outcomes. The reflection discussions also ensured that multiple perspectives were represented. Summaries of the workshop activities and key take-aways were provided to community partners. Follow up interviews were conducted with EPA Regional staff and community partners after one year for the Grand Rapids and Central Savannah River Corridor case studies to understand the outcomes and impacts of the workshops. The standardized note-taking, reflection discussions, and researcher field notes kept throughout the process of planning and executing the workshops provided quality assurance for qualitative data collection (Reynolds *et al* 2011).

For each case study, the notes from workshops, reflection discussions, and follow up interviews were transcribed and coded (in NVivo software) using deductive and inductive approaches (Cope 2010). Memoing, a common qualitative analysis method for self-reflection, was used to highlight points that needed clarification or further discussion with the entire research team and provide a record of the coding process. Further for quality assurance, each document was coded by two investigators, who then compared their codes to determine code norming (Reynolds *et al* 2011). A summary report with key findings and takeaways from the workshops will be released by EPA in 2024 (Eisenhauer *et al* in process).

## Results

In this section we share key themes from the analysis of the case study data focusing on lessons learned about how the workshop activities did or did not serve to advance the goals of community partners for equitable resilience. In addition, we highlight some less tangible aspects of participatory planning that are valuable and necessary for equitable resilience work such as the power of convening, recognizing trauma, and capacity for sustained engagement. These findings provide insights into how participatory engagement can generate discussions and ideas that contribute to equitable resilience planning efforts.

### Resilience and equity concerns

The workshop activities generated discussions about issues and hazards that threatened community members’ well-being and affected their ability to withstand or recover from disasters or other shocks and stressors. The ERB tool takes an ‘all-hazards’ approach which does not limit discussion to issues traditionally thought of as environmental or climate related but includes any issues that community members perceived as important threats to equitable resilience. In the workshops, participants described a variety of environmental hazards, disasters, and more social and health concerns.

The issues raised ranged from weather and climate change impacts such as flooding and heat, to environmental hazards such as water quality, to health and social concerns such as mental health problems and gun violence. Water quality was raised as a concern in many of the workshops. For example participants in the Central Savannah River Corridor noted recent changes in smell, taste, and color of water coming out of the faucets. Some residents spoke of their experience switching to bottled water usage, while others were unable to do so due to access and financial reasons. They feared this opened them up to exposure to contamination and potential health issues. They noted differences among geographic locations, with residents in Blackwell, SC, identifying water quality being worse near factories or along certain roads. While some residents were struggling for access to clean water, others had no idea this was even an issue in the area. Thus disparities across locations, and lack of access to information and resources, affected how participants were able to deal with exposure to a potential hazard causing stress and undermining the community’s resilience.

Many social issues were raised in the workshops as well, such as lack of opportunities for youth, gun violence and mental health issues. These reflect a concern with the higher risk and lower quality of life experienced by workshop participants that was felt by many to be the result of systemic racism and under-resourcing—the same underlying causes of environmental injustice and exposure to environmental hazards. Environmental justice scholars have long noted an understanding of the environment that is broader than the mainstream view primarily concerned with conservation of ‘nature’ and reflects a rights-based approach similar to other struggles for justice such as the civil rights movement (Taylor 2000).

These discussions provide information about resilience and equity issues not necessarily reflected in other local planning documents or sources of information on hazards. In Barnwell, SC for example, the EPA team reviewed existing planning documents and sources of information on environmental hazards including the Barnwell County Hazard Mitigation Plan, the National Climate Assessment Southeast chapter, NOAA’s Climate Explorer, FEMA’s National Risk Index, and NOAA’s Billion-dollar Weather and Climate Disasters. The hazards assessed in these sources include heat, drought, wildfire, flooding, winter storms, tornadoes, and earthquakes. They do not discuss equity issues or differential vulnerability of particular population groups, nor do they include issues like food security, homelessness, violence, water quality, and water supply that were raised by workshop participants.

### Social vulnerability

In many cases resilience planning includes social vulnerability assessments that identify sectors of the population which are particularly vulnerable to shocks and stressors from climate change or other hazards, disasters, and threats. These assessments are typically based on analysis of demographic factors such as age, race, language, income, disability which have been shown to represent population groups that are more likely to be exposed to or experience harm from hazards and disasters. However, these assessments have a number of important flaws and gaps (Painter *et al* 2024), particularly lack of data on some of the most marginalized and vulnerable groups such as LGBTQ+ individuals (Goldsmith *et al* 2022), undocumented immigrants (Maldonado *et al* 2016, Méndez *et al* 2020), and unhoused individuals.

In the ERB case study workshops we included a participatory social vulnerability assessment activity as a way of addressing some of these gaps. However, the activity did not fully achieve the desired results, demonstrating some important findings about the limitations of social vulnerability framing. The activity was conducted after the storytelling and participatory mapping activities, and introduced as a way to better understand the impacts of disasters on people in the community and why some can be more impacted than others. The facilitator asked participants to reflect on the two previous exercises and which groups were mentioned as being impacted. Then participants were asked to add other groups they believed might be particularly impacted and discuss the specific ways these groups might be impacted, and underlying reasons for those impacts. In three of the four workshops the activity yielded only minimally more information than in the previous two activities. Participants felt they did not have enough knowledge of how different groups might be impacted, and in one workshop a participant expressed concern that differentiating among groups in this way amounted to ‘labelling’ people which was objectionable. In one workshop, the activity did yield substantial discussion and new information—however in this case the team received feedback from a participant that the activity felt very ‘deficit focused.’ These findings align with the emerging literature that problematizes the use of the term ‘vulnerability’ in this way as stigmatizing and reducing the agency of individuals or groups (Munari *et al* 2021, Marino *et al* 2023). Based on this feedback the team discontinued the social vulnerability activity in further workshops.

### Equity and underlying social systems

The workshop activities brought out discussions of inequities in the ways that people were impacted by and the responses to hazards, disasters and threats, as well as the underlying reasons for these disparities. After the small group storytelling facilitators asked, ‘*What are similarities and differences in what people have experienced? What do we notice about the reasons for different impacts and experiences?’* The storytelling and mapping activities elicited discussions of different experiences among demographic groups such as age and income level, for example in how lower income households have more difficulty evacuating or may not be able to afford housing with a basement that provides protection during tornadoes. Participants also noted geographic differences such as between urban and rural, when for example rural households are more likely to have generators for power in the aftermath of a disaster and so restoring power is less urgent than in urban areas, but that rural areas may have greater need for debris cleanup.

In one workshop disparities among different zip codes within urban areas was mentioned early on, such as lack of storm drain maintenance in some neighborhoods that leads to greater risk of flooding. Several participants who noted that they paid close attention to local government decision making attributed the disparities to differences in income levels leading to higher tax revenue and more power for wealthier areas to influence conditions in their neighborhoods. This led to discussion of lack of resources and funding in lower income neighborhoods, and the lack of awareness of budgeting and decisions being made about how resources are spent. They attributed lower levels of involvement by lower income residents in decision making to inaccessibility of these processes due to factors such as meetings being held during daytime working hours and information being shared in a language that was not accessible to those directly impacted. In this way the discussion of disparities yielded multiple layers of underlying causes stemming from power imbalances and inaccessibility of public decision making.

In some cases facilitators were key in drawing out discussion of the underlying causes by asking what were the reasons for the differences. This elicited a wide range of underlying causes, from living a long time in a community and having strong personal connections close by who can help during a disaster, to having access to information technology to be able to get information during a disaster or apply for relief afterward, to lack of resources or political power. The group discussions prompted participants to make further connections with their own experiences. When one participant noted lack of access to information as an important reason for differential impacts, another participant who was a nurse then commented that she had noted language barriers as a factor in access to health care. In this way the stories were a catalyst for discussion, but facilitation prompted both broader and deeper discussion.

The storytelling and mapping discussions elicited rich knowledge about and experiences with the nature of local hazards, disasters, and threats, but what participants chose to focus on for identifying actions more often had to do with the underlying systemic issues, particularly social and health issues, than with specific climate or environmental hazards (table 1). Lack of access to communication and information was selected the most often, in four workshops. Other top social and health concerns were supporting the capacity of non-profit organizations, mental health and gun violence, housing and economic issues. Among climate and environmental concerns were flooding and stormwater, water quality, pollution and trash, and concern for and education on environmental issues. The emphasis on issues such as lack of information and resources shows that participants are well aware of how inequities that undermine resilience are systemic societal issues rather than simply about exposure to hazards. For example one group that discussed communication noted that organizations trying to share information need to make sure it is understandable to people they are trying to reach. As one participant said, ‘If there’s not a lot of engagement it’s because people don’t understand. They need to break it down so all people understand. A lot of people really care about climate change but they need to break it down to why this matters. You can’t just talk about elements that no one understands.’ Similarly in other workshops participants discussed plans to create resilience hubs and ecology education centers that would increase access to information and resources, and improve communication systems through text messages that would share information about services and funding opportunities to increase accessibility.

Even when actions were discussed without an apparent equity component, facilitators were able to draw out suggestions for making actions more equitable. For example, in one group that was discussing ways to reduce flooding, rain gardens and planting trees were suggested as potential ways for reducing runoff that impacted downstream communities. When a facilitator asked to make this equitable one participant mentioned organizing a rain garden installation in a local park but that they had to buy the plants, and that ‘we have to make these things not resource stressing.’ Another suggested that, instead of buying plants, cuttings and knowledge of how to propagate them could be shared with others, exemplifying the recognition that access to knowledge and sharing resources are meaningful actions for strengthening resilience.

### Power in convening

Opportunities for community building are an important part of the ERB process, in recognition of the fact that resilience is fundamentally relational. Stronger networks improve resilience in several ways. In the aftermath of disasters people frequently rely on networks of friends, family, and neighbors for assistance with basic needs and recovery (Misra *et al* 2017). Social capital, community cohesion, and networks are also especially important in under-resourced communities in everyday life, and strong networks and cohesion before a disaster contribute to greater well-being and reduced vulnerability overall (Patterson *et al* 2010, Sadri *et al* 2018). Additionally, networks are the foundation of political power and a community’s ability to effectively resist environmental injustices (Pastor *et al* 2001).

For this reason, holding workshops with community members lies at the heart of the ERB process. Convening workshops requires significant time and resources from conveners and participants however, and they are not always feasible or necessary. This is particularly true when information about community concerns already exists from previous planning efforts or research by community-based organizations. The ERB workshops were in-person however recent improvements in virtual meeting technology since the COVID-19 pandemic may make virtual workshops a viable option, particularly for rural communities.

We found distinct benefits to convening workshops, including fostering new and stronger connections, shared learning, and a sense of empowerment to take action. People shared information, knowledge, and contacts. One local organizer commented that the workshops brought together people from the community who didn’t previously know each other, or who knew of each other but had never come together to discuss these issues before. Participants encouraged each other to talk to their neighbors and create community with those around them to share information about what is going on in the community and to check in on others who might need assistance after disasters.

The workshops provided an opportunity for participants to hear from each other about issues affecting the community, share different perspectives and discuss potential actions. Some commented that they learned about issues they had not known about before that affected different parts of the community such as gun violence or access to drinking water. They also exchanged information about community events, available resources, and knowledge such as local programs to assist with rainscaping and tree planting. One local organizer reminded participants that ‘information is power, and we want to be powerful people.’

The workshop activities prompted thinking about collective action and steps they could take to improve resilience. Participants spoke about the importance of being active and participating in the community by volunteering, attending meetings, seeking project funding, speaking to neighbors and councilmembers, and voting. They generated ideas for collective action ranging from finding funding to establish a heating center, to creating a phone tree to stay in contact and check on neighbors in an emergency, to establishing a regular ‘food group’ for organizations fostering food sovereignty to meet and coordinate actions. They articulated values of working together and ‘plugging in’ to your network - including residents, community-based organizations and also local government leaders.

The act of convening brought government officials and community-based organizations together in the same room, which signaled to community members that government agencies are interested in understanding and addressing their concerns. The presence of local officials especially provided critical connections. One community-based organization shared their experience of frustration with the lack of responsiveness from city government to their requests made over several years for access to publicly owned vacant lots to establish community gardens. Within a week after the workshop, the organization received a call from city officials and were able to discuss a plan for moving forward. This demonstrates why building relationships between government officials and communities and local organizations catalyzes meaningful action that is responsive to community needs.

### Trauma

Although disasters are known to frequently cause trauma, the case studies demonstrated that a range of types and causes of trauma are relevant to resilience planning. According to the U.S. Substance Abuse and Mental Health Services Agency (SAMHSA 2024), trauma can stem from ‘an event or series of events experienced as harmful or life-threatening’ resulting in chronic effects on an individual’s functioning, as well as their social, physical, mental, spiritual, or emotional well-being. Trauma can result from the life-threatening and life-altering experience of disasters, but also from traumatic events such as the racially motivated mass shooting that occurred in the case study community of Buffalo, as well as the experience of chronic stress due to generational and systemic injustice, such as marginalization and exposure to environmental hazards.

Generational trauma can be understood through Kira’s (2001) taxonomy of ‘historic trauma’, defined as ‘a collective complex trauma [transmitted across generations] as it is inflicted on a group of people that have specific group identity or affiliation to ethnicity, color, national origin, or religion’ (80). Evidence of this trauma appears to have manifested in several workshops as participants mentioned feelings of distrust and lack of safety while interfacing with historically powerful groups, specifically white individuals, due to historical events such as broken treaties, Jim Crow systems, exploitive research experiments (Tuskegee), gentrification and displacement, and disproportionate environmental hazards in disadvantaged communities. Marginalization is inextricably linked to systemic traumas and was described by workshop participants in terms of political corruption, disparate justice, exclusionary practices, lack of resources and access, and mental health impacts, many of these occurring at the same time. One facilitator described the compounding effects of generational trauma and marginalization as ‘domino impacts’, with some aspects (e.g., Jim Crow systems) exacerbating others (lack of access, mental health impacts).

One case study community experienced acute trauma in addition to generational trauma, when a racially motivated mass shooting occurred at Tops Supermarket in East Buffalo in May 2022^7^, the ERB team hosted a training on trauma-informed approaches for our team members and community-based organization leaders, facilitated by the Institute for Diversity and Equity in Emergency Management. Discussions during the training were highly valuable to understanding experiences of marginalization such as being excluded from decision making processes or seeing promises broken that contributed to the lack of trust and created barriers to effective engagement with the community. Some expressed fears that the influx of funding would disappear as had happened in the past, or that it would lead to gentrification and displacement. Suggestions included increasing representation among those reaching out to communities since marginalized folks voiced that they were more likely to trust those reaching out if they looked like them: ‘…we want to talk to a Black person, we don’t want to spend time to build trust.’ Participants also voiced their appreciation for acknowledging trauma and historical mistakes that have been made with regard to power differentials. EPA and similar federal agencies were encouraged to be open and use mistakes as an opportunity to learn and bond: ‘Apologize and it shows you are human.’

### Capacity for sustaining engagement

Local organizers felt the workshops were a success in terms of their immediate goals for bringing together community members, deepening understanding of issues of concern and identifying potential actions to increase resilience and equity. They provided an opportunity to ‘think about projects differently, for example thinking beyond just water quality to more comprehensive injustices.’ Local partners followed up by sharing summaries of the workshops back with participants by email, integrating information from the workshops into a watershed resilience plan, holding follow-up calls with local leaders, and working to identify funding sources to continue resilience work. However sustaining engagement proved difficult without dedicated resources. One community partner noted that ‘there was some momentum we could have built on, but we didn’t have the capacity to follow up.’

This reflected perspectives expressed in the workshops about lack of access to resources in underserved areas and for community-based organizations (particularly smaller organizations) that are working directly in communities. Without sufficient resources organizations are limited in the amount of staff and time to participate in partnerships or initiatives beyond their primary mission such as resilience planning. Some participants representing CBOs noted that they were not aware of funding opportunities in their communities that should have been accessible to them and called for better communication and coordination from funders.

## Discussion

The ERB tool provides a set of participatory activities, guidelines, and principles within a process framework that can help improve incorporation of equity in resilience planning. Recognizing the limitations that many practitioners are starting from, and that it is impossible to anticipate every potential challenge that may occur during a planning process, these case studies illustrate that responding to challenges requires flexibility, listening to partners’ and community members’ feedback, and willingness to adjust are key to success. Each case study had unique goals, context, and timelines. Flexibility was particularly important for customizing the workshops for each community’s needs, goals, and timing and capacity constraints. In Barnwell and Waynesboro, workshops were held on Saturday to avoid interfering with participants work schedules. The community partner felt that full day workshops would be too taxing for participants and interfere with other responsibilities, and so the team decided to hold two half-day workshops instead. These were held two weeks apart which made it challenging for the team to finalize preparation of materials but felt necessary to maintain interest in participating and continuity of the workshop discussions. In Grand Rapids, adjustments were made between and within workshops to improve the experience for participants. As mentioned, the participatory social vulnerability analysis was discontinued after two workshops produced negative feedback, albeit different types. This had the benefit in the third workshop of allowing a slower pace for the activities and a more relaxed atmosphere. The team also noted that participants were interested in learning more about the watershed, and so the second and third workshops devoted more time to a ‘Watershed 101’ presentation and question and answer session. Agendas were also adjusted during the workshops to allow time for community building activities such as an ‘open mic’ session requested by participants in one workshop to share songs and spoken word, and in another case to visit a rain garden at the library where the workshop was held.

In Buffalo, traumatic events prompted the most significant changes in terms of the project timing and goals to respond to the new reality and capacity constraints of community partners. This also proved a valuable learning opportunity about the need for understanding and addressing trauma experienced by communities due to acute events such as violence and disasters, as well as trauma due to historic and long-standing marginalization. Instead of convening four workshops to cover the whole ERB process, the project began instead with an expert led training and discussion on trauma informed engagement. This allowed the ERB team and community partners to learn about different types of trauma and how they can be addressed and the importance of acknowledging the trauma that stems from a history of disinvestment and other forms of marginalization as a first step to building trust. The training also created an opportunity to hear from community partners about what the ERB team could provide that would be most helpful to them. They shared that there was likely fatigue in the community about storytelling and talking about needs, pointed the team to existing reports documenting community needs and concerns, and that the greater need was for more communication and coordination about funding sources that were available or coming to Buffalo but that community-based organizations and residents weren’t aware of or didn’t have access to. This became the focus of the next workshop which brought together representatives from local and state government, social services, community-based organizations, and foundations to brainstorm ideas and next steps. In this way the team adopted a ‘trauma informed approach’ in order to ‘avoid the causation of harm by re-traumatizing communities [and] better understand community needs’ (Rosenberg *et al* 2022). This also led to the development of a more in-depth resource on trauma informed approaches in the ERB tool in order to help planners better understand the effect trauma can have on participants and engagement. The storytelling activity was also revised to include techniques for recognizing potential trauma and conducting the activity in a manner sensitive to it.

Additionally, activities and resources in the ERB on workshop facilitation, community engagement and project planning resources build in flexibility, empathy and listening to provide support for anticipating and addressing challenges. For example, the core team leading the project should be inclusive and representative of the community, which will help ensure that community needs are heard and addressed. A discussion about barriers to participation provides an opportunity to identify and address potential barriers and enhance inclusivity. In the case study workshops A reflection diary provides opportunity for guided reflection at key points in the process to ensure that goals are being met or reevaluated as necessary and adjustments are made for potential challenges. Guides for implementation planning, monitoring progress, and maintaining engagement serve as a starting point for converting the data and information gathered and priorities expressed into action, with adaptation needed for each unique context.

The case studies make a number of contributions to the growing literature on equitable resilience planning. Historically, equity planning and resilience planning have overlooked each other, but there is a need to integrate equity into resilience planning, and vice versa, to promote truly sustainable and resilient communities (Meerow *et al* 2019, Mohabat Doost *et al* 2023). Typically planning processes conduct public involvement through techniques such as public meetings or comment periods or even more extended workshops or charettes, but these often fall short of what is needed and possible with more participatory, equity focused approaches: capturing intersectional inequalities, bringing forth nuanced social-environmental contexts, forming new partnerships, incorporating local voices and experiences with climate change, rectifying power inequalities, and building adaptive capacity (Amorim-Maia *et al* 2022, Jurjonas *et al* 2020, Rudge 2021, Berke *et al* 2023, Swanson 2023, Sarang and Prabhakar 2023). In contrast, participatory approaches used to achieve equity and justice include community partnerships and equity advisory boards (Diezmartínez and Short Gianotti 2022); empowering communities, mentoring local leaders, and representation of minority-focused organizations (Amorim-Maia *et al* 2022); engaging community groups to evaluate options and make decisions and using a trusted-advocates model (Berke *et al* 2023); citizen advisory boards and community visioning workshops (Cannon *et al* 2023); youth engagement, and citizen science (Fiack *et al* 2021). While the literature provides examples of how cities have utilized these approaches it doesn’t necessarily proffer specific methodologies for putting these into practice elsewhere. Moreover, these processes tend to be based on outreach from those in power instead of on innovative approaches for collaborative decision-making (Chu and Cannon 2021).

The process of engagement of historically marginalized groups is essential to equity and justice in resilience planning and implementation (Jurjonas *et al* 2020, Diezmartínez and Short Gianotti 2022). These case studies show how community engagement through participatory activities with a focus on equity can bring important perspectives and knowledge about local resilience challenges and underlying, systemic causes of vulnerability that is not available through existing data sources and is often left out of climate change planning as reviews of existing local and state resilience plans have demonstrated (Meerow *et al* 2019, Chu and Cannon 2021, Diezmartínez and Short Gianotti 2022, Raub *et al* 2023). We show how qualitative analysis of participatory processes illuminates what works to encourage inclusion and respectful dialogue, what doesn’t, and why, which allows lessons to be drawn that can inform future resilience planning efforts. There are challenges in bringing insights from the social sciences into climate adaptation and resilience planning, though. The climate policy-making process is not well suited to utilize input from the social sciences (Devine-Wright *et al* 2022). The scientific literature on adaptation has not kept pace with analyzing how equity and justice are being put into practice (Coggins *et al* 2021).

This research supports a growing understanding of resilience that emphasizes social dimensions and systemic inequity along with risk from climate and environmental hazards. Workshop discussions about community members’ concerns and desired actions tended to emphasize barriers to resilience that were related to access to resources and information, which are crucial for being able to make and carry out decisions that reduce risk and vulnerability. Issues such as access, once understood, can be actionable measures of equitable resilience (Logan and Guikema 2020). Incorporating social dimensions and systemic issues that underlie inequities is needed to ensure that resilience planning does not perpetuate injustice through reinforcing existing structural inequities (Jurjonas *et al* 2020, Zoll 2021).

## Conclusion

The growing literature on resilience planning makes clear calls for greater inclusion of justice and equity dimensions in resilience planning through participatory practices. We find that practices which facilitate inclusive discussions that intentionally address equity concerns bring community voices more strongly into planning activities for identifying actions to increase resilience. Indeed, without considering the experiences of community members, it is impossible to address the multifarious and complex sources of inequity and ways it hinders resilience across racial, gender, disability, socioeconomic and other intersecting lines. The Equitable Resilience Builder tool helps bridge the equity gap in resilience planning by building on decades of planning and other social science scholarship that shows that context matters (Ma *et al* 2023); the tool provides a practical guide for designing flexible and responsive engagement processes with participatory planning activities to include community voices. Such tools provide important advances beyond social vulnerability assessment methods whose limitations are receiving increased attention (VanBuskirk and Mullenbach 2024). Reducing vulnerability is not achievable without understanding and addressing system factors such as marginalization and underinvestment in impacted communities. A pivot from a social vulnerability framing to equitable resilience can allow for inclusive, participatory, locally grounded knowledge to inform planning in ways that are empowering rather than deficit focused.

There is much more to be learned about how participatory planning ultimately improves resilience, given the newness of the field and lack of longitudinal research. But in the short term the results of these case studies demonstrate that a participatory, flexible and responsive process yields a deeper understanding of equity, social and health concerns in a community, a sense of empowerment, and stronger networks and social capital, all of which are important for resilience (Misra *et al* 2017, Sadri *et al* 2018, Pastor *et al* 2001, Patterson *et al* 2010). We examine the utility of narrative storytelling and other qualitative approaches to engaging key participants and assessing hazards as more humanizing methods of gathering data that provide an opportunity for community members to share perspectives on the underlying systemic issues such as lack of access and resources that underlie inequities. We highlighted the value of convening to bring together community members, leaders, and government officials and foster a sense of empowerment. We noted the importance of acknowledging historic trauma to demonstrate responsiveness and begin to build trust.

Facilitation, capacity and lack of resources to support participatory and inclusive processes is still a major barrier to equitable resilience planning however. This is particularly true when it comes to sustaining engagement with community members and community-based organizations or funding the eventual implementation of actions that community members have called for. Without dedicated support, equitable resilience work becomes less feasible, and so it is important for funders to understand the value of supporting inclusive resilience planning that centers equity through meaningful and sustained engagement.

## Figures and Tables

**Table 1.** Top concerns selected by workshop participants for brainstorming actions.

Social or health concerns:	Climate or environmental hazards:
Lack of access to communication, information, or broadband (4)	Flooding and stormwater runoff (2)
Lack of resources and capacity support for non-profit organizations (2)	Water quality (2)
Gun violence and mental health (2)	Pollution and trash (2)
Health and drug use/overdoses (2)	Concern for the environment and environmental education (2)
Homelessness/economic disparity and housing/workforce development (2)	Chemical releases
Transportation	Heat
Cyber threats	Storms
	Lack of access to natural areas and recreation
	Riverine water levels and temperature for biodiversity

## Data Availability

The data cannot be made publicly available upon publication because they are not available in a format that is sufficiently accessible or reusable by other researchers. The data that support the findings of this study are available upon reasonable request from the authors.

## Footnotes

6 Climate Resilience Consulting, Adaptation International, and Kim Lundgren Associates.

7 Artemis Moshtaghian, Emma Tucker, Shimon Prokupecz and Samantha Beech. ‘10 people killed in a racially motivated mass shooting at a Buffalo supermarket, police say. The 18-year-old suspect is in custody.’ CNN, May 14, 2022. Accessed on-line at: https://edition.cnn.com/2022/05/14/us/buffalo-ny-supermarket-multiple-shooting/index.html.
